# Personality traits affect anticipatory stress vulnerability and coping effectiveness in occupational critical care situations

**DOI:** 10.1038/s41598-022-24905-z

**Published:** 2022-12-05

**Authors:** Sophie Schlatter, Simon Louisy, Brice Canada, Corentin Thérond, Antoine Duclos, Chris Blakeley, Jean-Jacques Lehot, Thomas Rimmelé, Aymeric Guillot, Marc Lilot, Ursula Debarnot

**Affiliations:** 1 Research on Healthcare Performance (RESHAPE), INSERM U1290, Univ. Lyon, UCBL-Lyon 1, Lyon, France; 2 Inter-University Laboratory of Human Movement Biology, EA7424, UCBL-Lyon 1, Univ. Lyon, 69622 Villeurbanne, France; 3 High-Fidelity Medical Simulation Centre (CLESS), SAMSEI, UCBL-Lyon 1, Univ. Lyon, Lyon, France; 4 Departments of Anaesthesia and Intensive Care, Hospices Civils de Lyon, Lyon, France; 5 Laboratory of Sport Vulnerabilities and Innovations, EA7428, UCBL-Lyon 1, Univ. Lyon, Villeurbanne, France; 6 Health Data Department, Hospices Civils de Lyon, Lyon, France; 7 EA7426 “Pathophysiology of Injury-Induced Immunosuppression” (Pi3), Biomérieux-Hospices Civils de Lyon, UCBL-Lyon 1, Univ. Lyon, Lyon, France; 8 Institut Universitaire de France, Paris, France

**Keywords:** Emotion, Stress and resilience, Human behaviour

## Abstract

The present study aimed at investigating the influence of personality on both anticipatory stress vulnerability and the effectiveness of coping strategies in an occupational stressful context. Following assessment of individual personality traits (Big Five Inventory), 147 volunteers were exposed to the anticipation of a stressful event. Anxiety and cardiac reactivity were assessed as markers of vulnerability to anticipatory stress. Participants were then randomly assigned to three groups and subjected to a 5-min intervention: relaxation breathing, relaxation breathing combined with cardiac biofeedback, and control. The effectiveness of coping interventions was determined through the cardiac coherence score achieved during the intervention. Higher neuroticism was associated with higher anticipatory stress vulnerability, whereas higher conscientiousness and extraversion were related to lower anticipatory stress vulnerability. Relaxation breathing and biofeedback coping interventions contributed to improve the cardiac coherence in all participants, albeit with greater effectiveness in individuals presenting higher score of openness to experience. The present findings demonstrated that personality traits are related to both anticipatory stress vulnerability and effectiveness of coping interventions. These results bring new insights into practical guidelines for stress prevention by considering personality traits. Specific practical applications for health professionals, who are likely to manage stressful situations daily, are discussed.

## Introduction

Stress vulnerability refers to the established predisposition of some individuals to have an elevated psychophysiological response to stressful stimuli. Stress vulnerability has many immediate effects, such as decreased wellbeing and increased risks of incidents, as well as long-term deleterious consequences (e.g., development of mental and cardiovascular disorders)^1–4^. Determining factors, such as personality traits, that may affect stress vulnerability is necessary to reduce these risks and promote individualized stress management training. Personality is reflected in a set of patterns of thoughts, feelings and actions^5^. The widely used Five Factor Model allows characterization of personality into five traits: neuroticism, extraversion, agreeableness, conscientiousness and openness to experiences^6^. Personality traits have been repeatedly associated with many psychophysiological markers of stress (e.g., cardiac reactivity, cortisol secretion, psychological stress)^7–15^. Neuroticism has been repeatedly linked to an increase in negative emotions and related physiological responses such as increased heart rate, cortisol reactivity and amygdala activity during a stressful event^13,15,16^. By contrast, extraversion and openness have both been associated with lower levels of subjective stress, lower cardiovascular reactivities and reduced cortisol secretions^7,9,15^. Findings for the conscientiousness trait remain inconclusive, being neither advantageous or disadvantageous in a stressful situation, while agreeableness remains, so far, less explored^7,13^. Based on these results, neuroticism may be associated with higher stress vulnerability, while openness and extraversion seem associated with reduced vulnerability (i.e. higher resilience).

Most of the investigations in the domain of stress evaluated the influence of personality during confrontation with a standardized laboratory stressor, limiting the generalisation of results to real-life stressors. Indeed, most paradigms used stereotypical stressors such as a mental arithmetic task, the Stroop task, an anger recall, or the Trier Social Stress Test, which do not optimally represent stressors typically encountered in everyday life^7,9,15^. Nowadays, ecological studies are crucial to understand in greater detail the relationship between individual characteristics and vulnerability to real stressors. Recent advances made it possible to overcome the limitations of standard laboratory tasks by implementing virtual reality or high-fidelity simulations (also called full scale simulations). Simulation environments enable situations still closer to real-life along with experimental control, hence allowing investigation of the direct effects of stress on professional performance^17–22^. Simulation studies usually focus on urgent and crucial professional situations, such as the management of critical care for anaesthetists^20–22^. These simulations remain indispensable for training anesthesiology residents to practice in one of the most stressful medical professions^23^.

In many daily-life contexts, stressful situations can be detected before the occurrence of the actual stressor. The stress anticipation induces immediate psychophysiological responses and influences the subsequent acute stress response^24–28^. Interestingly, the period of stress anticipation offers a window of opportunity to benefit from a preventive coping intervention^20,24,25^. Several and various coping techniques including relaxation breathing, cerebral stimulation, or biofeedback have been explored to manage threat, harm, or stress^20,24,25,29,30^. Among these interventions, relaxation breathing appeared the most robust option for managing stress reactivity, due to its cost-effective nature and its ease of implementation in almost all stressful situations^20^. When paired with cardiac biofeedback, relaxation breathing resulted in a significant reduction of psychophysiological stress markers notably during anticipatory stress^20,24,25,31–34^.

Successful management of anticipatory stress may depend on the interaction between individuals’ characteristics and the implemented coping intervention. Previous studies revealed that personality traits are likely to modulate the effectiveness of coping methods such as biofeedback, brain stimulation, muscle relaxation, or meditation^30,35–37^. To date however, no study has investigated the moderating role of personality traits in the effectiveness of relaxation breathing and cardiac biofeedback. Determining in greater detail the relationship between personality and coping intervention effectiveness may allow design of relevant and individualized programs for the prevention of stress related disorders.

The present study therefore aimed to explore the association between personality traits and both anticipatory stress vulnerability and coping intervention effectiveness. This study characterises the influences during the anticipation of real-life stressful situations, such as medical critical care situations, by using high fidelity simulation. We hypothesized that higher neuroticism and lower extraversion traits may be related to anticipatory stress vulnerability. We further expected that personality would influence the effectiveness of relaxation breathing with or without a cardiac biofeedback intervention.

## Materials and methods

### Participants

A total of 147 participants voluntarily took part in this experiment (42 women, 26.8 $\pm$ 2.3 years old). Volunteers were recruited during their training in critical care scenarios at the medical simulation centre of Lyon University during the academic year 2019–2020. Participants were all anesthesiology and critical care residents (from the first to the fifth year). Four investigators provided information about the study, collected written individual informed consent, and enrolled participants. The study was approved by the Institutional Review Board of Claude Bernard University Lyon 1 (Lyon, France, IRB 2019070903) and informed written consent was obtained from each participant prior to inclusion. All experiments were performed in accordance with the Declaration of Helsinki.

### Personality

At the beginning of the experiment, all participants filled-out a demographic questionnaire and the French validated version of the Big Five Inventory (BFI-fr)^38^. The BFI contains 45 items assessing openness to experience, conscientiousness, extraversion, agreeableness and neuroticism (Table 1). Each item assesses agreement or disagreement with descriptive statements on a 5-point Likert scale ranging from 1 (strongly disagree) to 5 (strongly agree). For each of the five personality traits, a mean was determined. In this study, the internal consistencies for each personality traits ranged from acceptable to good (openness to experience α = 0.66, conscientiousness α = 0.77, extraversion α = 0.88, agreeableness α = 0.78, neuroticism α = 0.84).

**Table 1:** The Table of definitions of the five personality traits.

Openness to experience	Being creative and open-minded	Tendency to be curious and unconventional
Conscientiousness	Being organized and responsible	Tendency to self-discipline and responsibility
Extraversion	Being sociable and energetic/active	Tendency to experience positive emotions and to be sociable
Agreeableness	Having compassion and willingness to cooperate	Tendency to be trusting and cooperative
Neuroticism	Being emotionally unstable	Tendency to experience distress and negative emotions

### Paradigm

**Figure 1:**
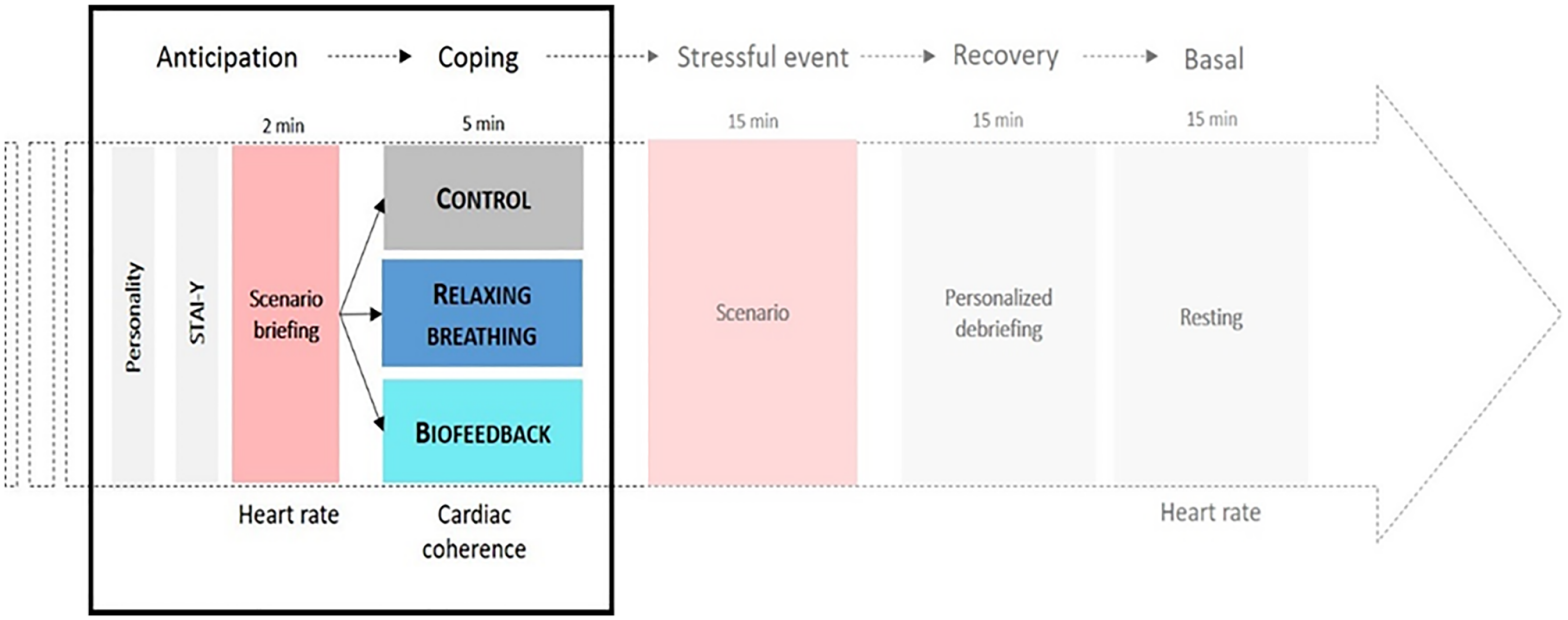
Protocol timeline. STAI-Y: State Anxiety Inventory form A.

All participants performed a high-fidelity (full-scale) simulation that was divided into five periods: scenario briefing (2 min, anticipation), coping intervention (5 min, coping), the stressful critical care scenario (15 min), personalized debriefing (15 min), and a final resting state period during which a collective debriefing occurred (15 min, basal). The present study focused on the anticipation and coping periods (Fig. 1).

### Anticipation period

To assess psychological anticipatory stress, all participants filled out the State Anxiety Inventory Form A (STAI-Y)^39,40^. In this study, the STAI-Y had an excellent internal consistency (Cronbach α = 0.94). Then, during the two minutes of the briefing, each participant received information on the scenario in which he/she would be involved (for more detailed information please refer to the Annexe 1). The high-fidelity simulation reproduces professional critical clinical situations through realistic scenarios chosen to fit to the expected competency level of residents.

To determine physiological anticipatory stress, a score of cardiac reactivity was computed (mean heart rate during anticipation *minus* mean heart rate basal) for each resident; a high score reflected high physiological stress^11^. Means were extracted from the continuous monitoring of cardiac activity (HexoskinTM, Carre Technologies Inc, Montreal, Quebec, Canada).

### Coping period

**Figure 2:**
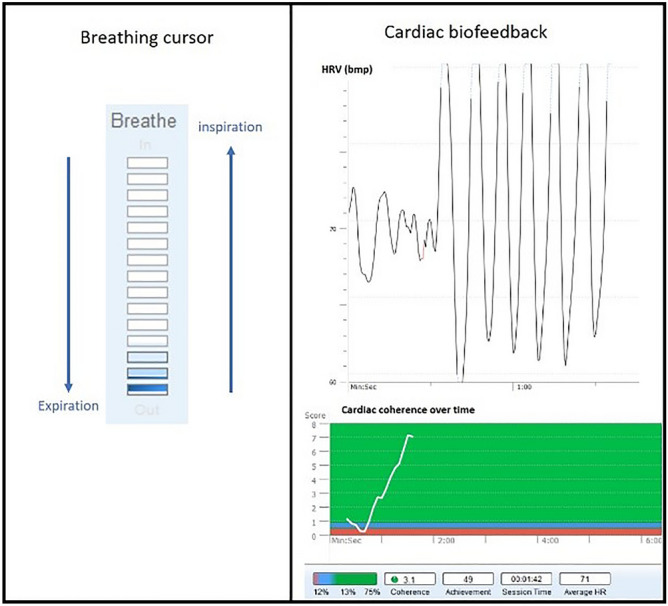
Coping interventions. Breathing cursor (left part) and cardiac biofeedback visual interface (right part). The visual cursor driving the inspiration and expiration at 6 breaths/min. The visual biofeedback gave information on instantaneous heart rate (beats/min) and scores of cardiac coherence (emWavePRO® interface, HeartMath technologies, Add Heart®).

Following the briefing, participants were randomly assigned to one of the three 5-min interventions. All the interventions were conducted in an isolated and silent room, and were all guided by the same experimenter (S.S). Participants in the relaxation breathing group (relaxation breathing) performed standardized relaxing breathing at 6 breaths/min with the help of a visual breathing cursor (Fig. 2, left part). Participants in the cardiac biofeedback group (biofeedback) performed the same breathing exercise, while visualizing information on their instantaneous heart rate (beats/min) and scores of cardiac coherence (Fig. 2, left and right) (emWavePRO® interface, HeartMath technologies, Add Heart®). The relaxation breathing and biofeedback interventions were displayed via a computer interface window of 5 × 2.8 inches. Participants in the control group (control) performed a common professional activity (i.e. reviewed printed laboratory test results). The duration of the intervention (5 min) was chosen to fit the numerous occupational-life contexts where time-constraints are important. A previous study showed that 5 min of relaxation breathing or biofeedback intervention appears sufficient to reduce psychological stress^20^.

An ear pulse sensor was attached to all participants to record the cardiac activity at 370 Hz (HeartMath technologies, Add Heart®). The cardiac coherence score was computed as (Peak Power / [Total Power − Peak Power]) on a min-by-min basis, this score is assessed and displayed by the EmWave software^41,42^. As a high cardiac coherence score has been associated with increased positive emotional regulation^25,43,44^, the score achieved during the intervention was used to determine individual coping ability, with high scores corresponding to efficient physiological stress coping.

### Statistical analysis

**Figure 3:**
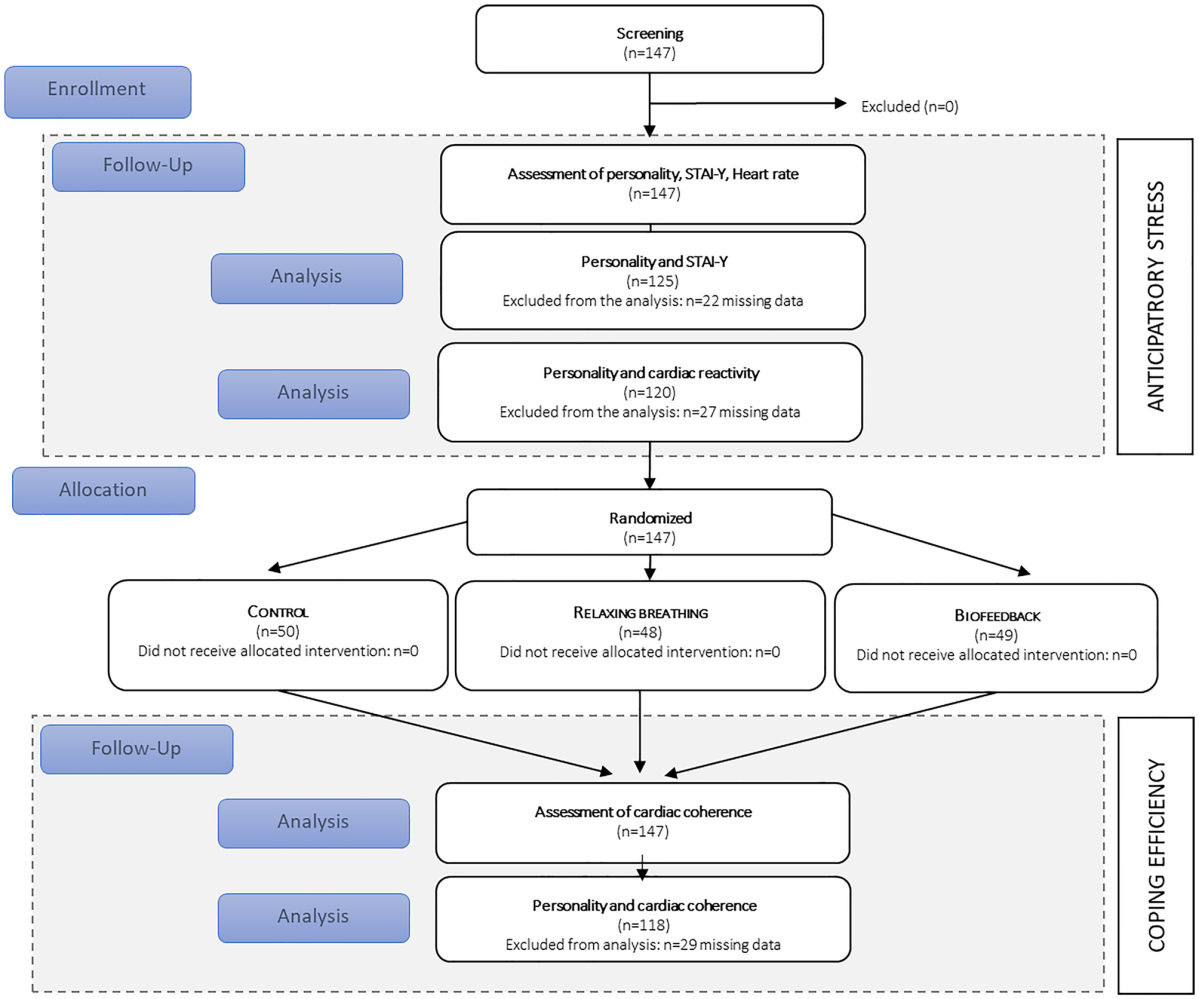
Study flowchart.

Linear regression analysis was used to predict psychological anticipatory stress (STAI-Y), cardiac reactivity, and cardiac coherence by personality trait. Each personality trait was analysed separately. The analysis was controlled for age, gender, weight, height and amount of sport practiced per week because these factors are likely to influence cardiac parameters and/or psychological stress responses^9,11,15^. The level of education was included in order to adjust for the probable influence of experience in stress management due to training in the medical curriculum. All quantitative variables of adjustment have been standardized. In the case of cardiac coherence, the interaction term (trait X group) was considered. Statistical analyses were performed with R studio version 1.2.1335 (R Foundation, Vienna, Austria). For all regression models, the β (i.e., estimate the effect on the outcome of each 1-unit increase in the independent variable) and the adjusted coefficients R^2^ (i.e., percentage of variance explained) were provided. Normality of residuals of the models were checked. Inclusions for analyses are shown in the study flowchart (Fig. 3).

## Results

### Cohort characteristics

Participants’ demographic and psychometric parameters are presented in Table 2.

**Table 2:** Participants demographical and psychometric parameters (n = 147).

	Mean ± SD or %
**Demographic parameters**	
Age (years old)	26.8 ± 2.3
Weight (kg)	69 ± 11
Size (cm)	175 ± 8
Sports per week (hours)	3.51 ± 2.54
Females (%)	28.57%
**Psychometric parameters**	
Openness (score)	3.32 ± 0.54
Conscientiousness (score)	3.75 ± 0.58
Extraversion (score)	3.08 ± 0.86
Agreeableness (score)	4.14 ± 0.53
Neuroticism (score)	2.77 ± 0.78

### Personality and anticipatory stress vulnerability

**Figure 4:**
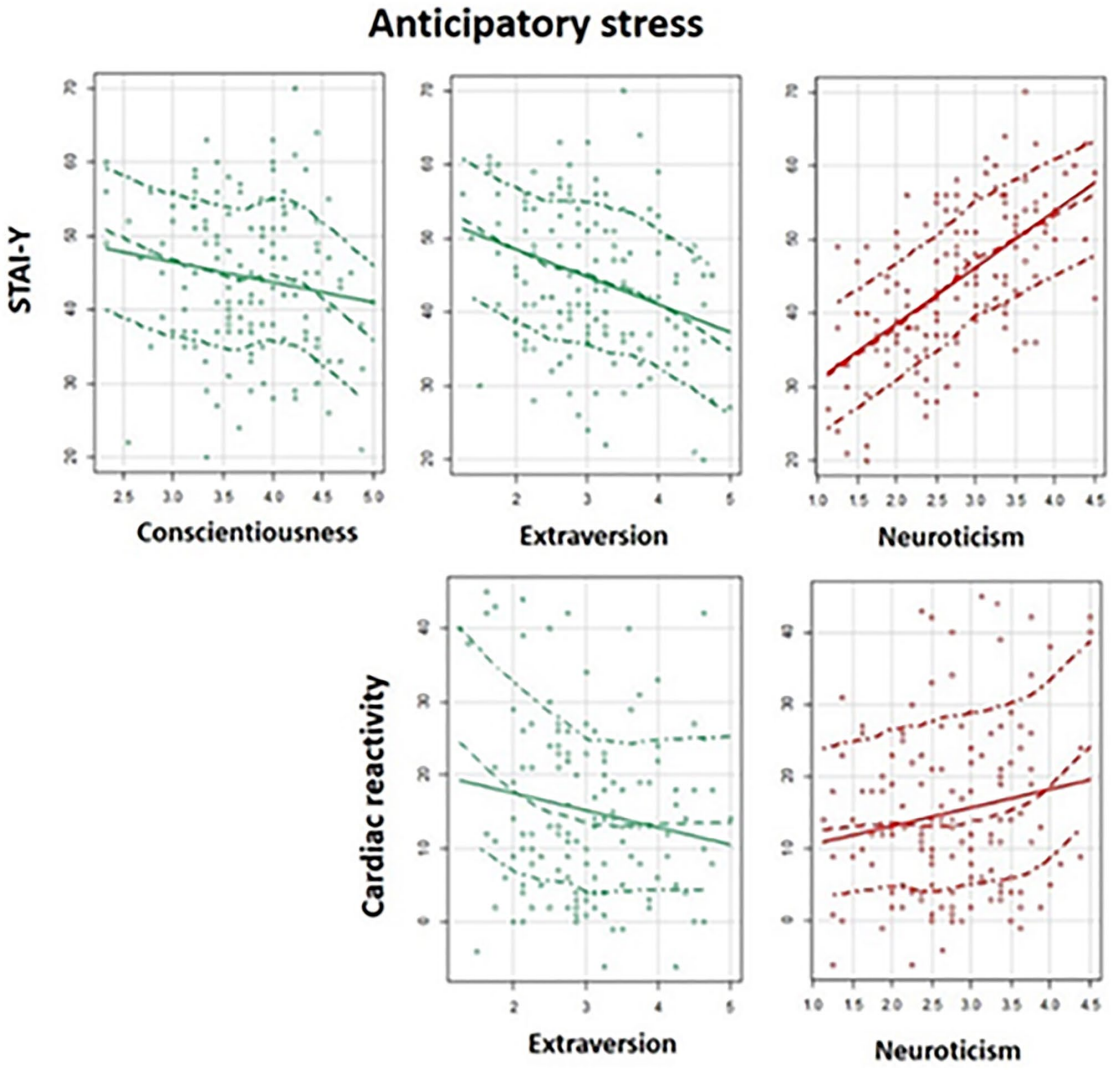
Personality traits and anticipatory stress. Association between personality traits and psychological (STAI-Y) and physiological anticipatory stress (cardiac reactivity). Green plots represent traits positively associated with a stress-resilience aspect of personality. Red plots represent traits that are positively associated with a stress-vulnerability aspect of personality.

A higher score of conscientiousness was associated with a lower level of psychological anticipatory stress (β = − 0.193, *P* = 0.036). A higher score of extraversion was associated with a lower level of psychophysiological anticipatory stress (β = − 0.300, *P* = 0.002; β = − 0.210, *P* = 0.029). However, a higher score of neuroticism was associated with a higher level of psychophysiological anticipatory stress (β = 0.631, *P* < 0.001, β = 0.229, *P* = 0.036) (Table 3, Fig. 4).

**Table 3:** Adjusted regression models for each personality trait and anticipatory stress (STAI-Y and cardiac reactivity) (n = 120).

	Standardised β	Standardised SE	*P*	Adjusted R^2^
**STAI-Y (Psychological anticipatory stress)**				
Openness	− 0.170	0.094	0.071	0.046
Conscientiousness	− 0.193	0.091	**0.036**	0.056
Extraversion	− 0.300	0.095	**0.002**	0.097
Agreeableness	− 0.046	0.098	0.636	0.021
Neuroticism	0.631	0.083	** < 0.001**	0.346
**Cardiac reactivity (Physiological anticipatory stress)**				
Openness	− 0.064	0.094	0.497	− 0.002
Conscientiousness	− 0.073	0.093	0.435	− 0.001
Extraversion	− 0.210	0.095	**0.029**	0.036
Agreeableness	0.170	0.095	0.078	0.022
Neuroticism	0.229	0.105	**0.036**	0.033

Significant relationships of the model analyses are in bold. SE is the standard error.

*STAI-Y*: State Anxiety Inventory.

### Personality and coping intervention effectiveness

**Figure 5:**
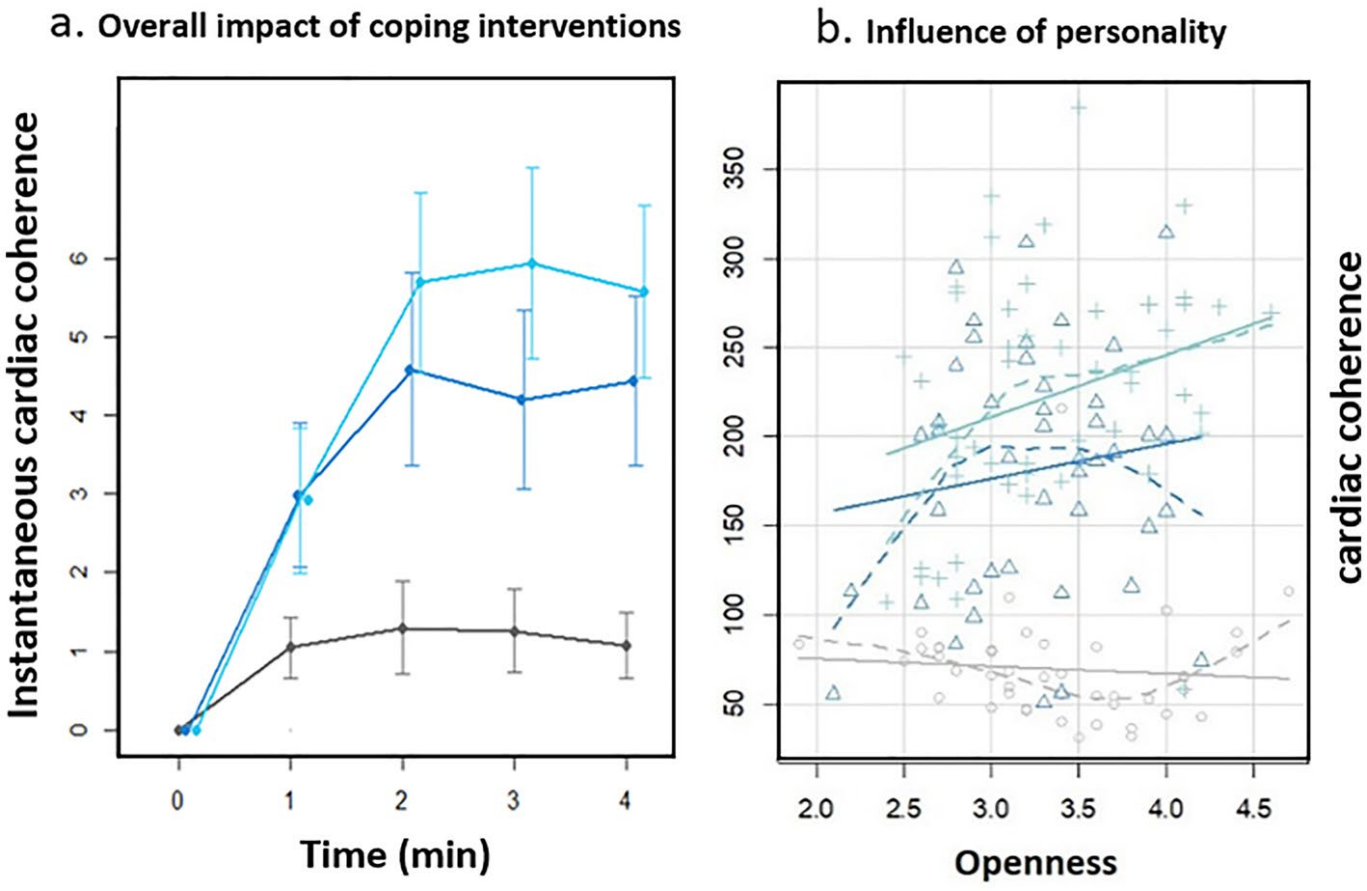
Effectiveness of coping interventions on cardiac coherence score. (**a**) Overall impact of relaxation breathing (dark blue), relaxation breathing paired with biofeedback (turquoise) and control (grey) on the evolution of the instantaneous score of cardiac coherence. Both coping interventions allows an increase in cardiac coherence scores. (**b**) Openness trait and cardiac coherence scores. Scatterplot of the relationship between openness and interventions. The grey circles indicate the control group, the dark blue triangles indicate the relaxation breathing group and the turquoise crosses indicate the relaxation breathing paired with biofeedback group. Higher score of openness are associated with higher score of cardiac coherence and so more efficient physiological stress coping (i.e. physiological relaxation).

Compared to the control, both the relaxation breathing and biofeedback induced an increase in cardiac coherence during the coping period (Fig. 5A). Higher score of openness was associated with a greater score of cardiac coherence in both relaxation breathing (β = 0.413, *P* = 0.010) and biofeedback (β = 0.284, *P* = 0.038) groups. No other personality traits were associated with the score for cardiac coherence (Table 4, Fig. 5B).

**Table 4:** Adjusted regression models for each personality trait and cardiac coherence (n = 118).

Cardiac coherence score	Standardised β	Standardised SE	*P*	Adjusted R^2^
**Openness**				
trait	− 0.088	0.100	0.385	0.633
relaxation breathing	1.361	0.144	** < 0.001**	
biofeedback	1.753	0.142	** < 0.001**	
trait x relaxation breathing	0.413	0.158	**0.010**	
trait x biofeedback	0.284	0.135	**0.038**	
**Conscientiousness**				
trait	− 0.018	0.102	0.859	0.608
relaxation breathing	1.302	0.146	< 0.001	
biofeedback	1.743	0.147	< 0.001	
trait: relaxation breathing	0.120	0.144	0.407	
trait: biofeedback	− 0.214	0.149	0.153	
**Extraversion**				
trait	0.055	0.100	0.583	0.599
relaxation breathing	1.284	0.149	< 0.001	
biofeedback	1.741	0.149	< 0.001	
trait: relaxation breathing	0.008	0.149	0.956	
trait: biofeedback	0.236	0.152	0.123	
**Agreeableness**				
trait	− 0.026	0.132	0.842	0.615
relaxation breathing	1.259	0.146	< 0.001	
biofeedback	1.798	0.149	< 0.001	
trait: relaxation breathing	− 0.205	0.161	0.206	
trait: biofeedback	− 0.104	0.182	0.570	
**Neuroticism**				
trait	− 0.010	0.115	0.933	0.590
relaxation breathing	1.299	0.150	< 0.001	
biofeedback	1.758	0.150	< 0.001	
trait: relaxation breathing	0.069	0.155	0.657	
trait: biofeedback	− 0.045	0.145	0.760	

Significant relationships of the model analyses are in bold. SE is the standard error.

## Discussion

The aim of this research was to determine the influence of personality traits on the anticipatory stress vulnerability and effectiveness of preventive coping intervention. This study characterised the influences of personality traits during the anticipation of a simulated professional critical care situation. Results showed that individuals with high scores of neuroticism (N^+^), low scores of conscientiousness (C^−^), or low scores of extraversions (E^−^) exhibited elevated anticipatory stress markers. Interestingly, openness was found to positively predict the effectiveness of relaxation breathing and biofeedback coping interventions.

As expected, results revealed that neuroticism was associated with worsened anticipatory stress vulnerability (i.e., N^−^ resilience, N^+^ vulnerability). Accordingly, a higher neuroticism score predicted elevated psychological and physiological anticipatory stress responses. Neuroticism is a higher-order personality trait that accounts for a tendency to experience emotional instability, distress and negative emotions^45,46^. We postulated that these tendencies led to a greater level of subjective stress, which in turn is manifested by elevated cardiac reactivity. Others studies found that N^+^ is linked to an increase of vulnerability during the confrontation with the stressor^13,15,16^ and with the long-term development of stress related-pathologies (e.g., burnout, depression)^47^. Present results therefore represent a new step in the overall understanding of the relationships between neuroticism and vulnerability with early stress anticipation, acute stress and mental disorders.

Our findings also revealed that extraversion was inversely associated with anticipatory stress vulnerability (i.e., E^−^ vulnerability, E^+^ resilience). Lower scores for extraversion predicted elevated psychological and physiological anticipatory stress responses. This result is consistent with previous findings reporting higher subjective stress and cortisol level in E^−^ individuals during the confrontation with a standardized stressor (mental arithmetic, anger recall, Trier Social Stress Test)^9,10,15^. Extraversion is a higher-order personality trait that accounts for an individual’s tendency to experience positive emotions and to be sociable^45,46^. In our experiment, we hypothesized that the tendency to experience positive emotions induced a more positive mindset for dealing with the situation, which turns into a lower level of psychophysiological stress. It is also possible that the tendency to be sociable resulted in a lowering of general anxiety and specific anticipatory stress. Indeed, social support is one of the main strategies used for coping or regulating cognition in response to stressors, as assessed by the Brief Cope Scale Inventory (social support, avoidance, positive thinking, problem solving)^48^.

While not predicted, our results also showed that conscientiousness was negatively associated with psychological anticipatory stress vulnerability (i.e., C^−^ vulnerability, C^+^ resilience). Literature reveals that individuals with a higher conscientiousness trait use problem-focused coping methods^49–51^. In the present work, the stressful situation was a multimodal complex task, where a high level of competence was required to succeed. It seems likely that C^+^ individuals focusing on problem-solving may have the higher level of theorical resources necessary to deal with certain aspects of the critical situation. Similarly, C^+^ individuals have an increased tendency to self-discipline and responsibility^45,46^. As an explanatory track, we hypothesized that a high-level of self-discipline might contribute to lowered levels of anticipatory stress notably by allowing individuals to have control over the situation. Taken together, our results revealed that individuals with a high neuroticism trait, and/or a low extraversion or conscientiousness trait, are the most vulnerable to an anticipatory stress, and confirm the importance of evaluating these traits for understanding stress responses.

For the first time, this study evaluated the influence of personality traits on the effectiveness of relaxation breathing and cardiac biofeedback interventions. Our data revealed that individuals with high scores of openness (O^+^) achieved better cardiac coherence scores. This is in agreement with Peciuliene et al. (2015) who found that individuals with high openness scores presented more physiological benefits (i.e., lowering physiological arousal) following biofeedback training based on skin conductance^30^. Others studies further revealed that openness was positively associated with perceived coping ability, notably during a stressful event, and a greater level of control over the task^7,52^. As both coping interventions tested here relied on breathing-control, it seems possible that the openness trait led to more effective practice of these new exercises, which in turn resulted in efficient stress reduction^53^. Furthermore, individuals with high openness trait are curious by nature^45,46^, which might make them spontaneously more interested in trying a new exercise. Future studies exploring various interventions are necessary to determine which technique is the most efficient coping method for N^+^, E^−^ and C^−^ individuals. Previous findings suggested that stress reduction through meditation is a promising alternative for N^+^ individuals^36^, while cerebral stimulation might be more effective for E^−^ individuals^54^. Comparing these alternative coping strategies using the same stressful paradigm will be necessary to confirm the specificity of the intervention-trait relationships. These studies are needed before offering preventive personalized treatment and to prevent the development of stress related disorders.

As a final practical observation, this study explored ecological stress within a medical professional context. In addition to the usual decrease of well-being and health, high levels of stress in physicians are associated with poor quality of care and increased risks of incidents^3,4^. Identifying a physician’s personality traits associated with anticipatory stress is relevant to estimate these professional risks. Our results showed that N^+^, E^−^ and C^−^ anaesthesiologists and critical residents are vulnerable to anticipatory stress. Previous studies repeatedly reported that N^+^ and E^−^ influenced level of school and job stress, and are associated with the risk of burnout^47,55,56^. Together, these results support that N^+^ and E^−^ anaesthetists are at great risk of both immediate and long-term specific professional stress, and should be prioritized to early stress coping training. Our findings suggest that relaxation breathing and cardiac biofeedback might contribute to increase scores of cardiac coherence in all profiles (even in N^+^ and E^+^ individuals), thus these interventions might be efficient to reduce physiological stress during the anticipation of a stressful critical care event. One should note that our study explored the influences of personality traits using high fidelity simulations, thus it remains necessary to duplicate our findings in a real-life hospital context. Replication of the present findings in other daily real-life settings (e.g. job interview, competitive sport) will also help to generalize our conclusions to other contexts. As a final remark, it would be interesting to perform similar studies comparing younger students and older professionals to examine if our findings are moderated by the individual’s coping experience, generated by repeated confrontation with stressful situations.

This study has some limits that should be emphasized in order to prevent over interpretation of the results. Firstly, while our finding paves the way for understanding the influence of personality on stress coping in an ecological context, it should be noted that both the stressor (i.e., anticipation of a critical care) and the population (i.e., anaesthesiology and critical care residents) remain specific. Therefore, results may not generalize to other occupational stress contexts, while generalization to other population remains speculative, the personality of our medical students appeared similar to the one of others students in other domains/speciality (see Annexe 2). Secondly, while conscientiousness, extraversion, agreeableness, and neuroticism present all Cronbach alpha scores superior to 0.70 and could be considered as satisfactory^38^, one should note that the consistency of the openness trait was a little lower (0.66). Thirdly, the percentage of variance explained by our models ranges from 3 to 60. While the coping effectiveness models explained more than 50% of the variation of cardiac coherence scores, the anticipatory stress models showed a low adjusted R^2^. Thus, the relationship between personality traits and anticipatory stress was relatively small. As anticipatory stress appears to be determined by multiple factors (e.g., influence of gender, experience, age, inner resources), each factor, including personality traits, is unlikely to have a large effect. Fourthly, due to the innovative and exploratory aspect of our study no correction for multi tests were applied, thus the error risk might be inflated.

To conclude, our results showed that individuals with a high neuroticism trait and a low extraversion trait are the most sensitive to anticipatory psychophysiological stress. Moreover, those with high openness to experience trait benefited more extensively from brief coping interventions. These data encourage the assessment of personality in identifying ecological stress vulnerability and offering individualized stress management training including relaxation breathing and cardiac biofeedback. Practically, these data offer a promising direction to pursue in investigating how to protect vulnerable health professionals from chronic stress.

## Supplementary Information

Supplementary Information.

## Data Availability

The datasets used and/or analysed during the current study are available from the corresponding author on reasonable request.

## Abbreviations

BFI: Big Five Inventory
BFB: Cardiac biofeedback
CI: Confidence Interval
FFM: Five Factor Model
HR: Heart Rate
RB: Relaxation Breathing
SD: Standard Deviation
HRV: Heart Rate variability

## References

[1] Chida Y, Steptoe A (2010). Greater cardiovascular responses to laboratory mental stress are associated with poor subsequent cardiovascular risk status: A meta-analysis of prospective evidence. Hypertension.

[2] Treiber FA, Kamarck T, Schneiderman N, Sheffield D, Kapuku G, Taylor T (2003). Cardiovascular reactivity and development of preclinical and clinical disease states. Psychosom. Med..

[3] Firth-Cozens J, Greenhalgh J (1997). Doctors’ perceptions of the links between stress and lowered clinical care. Soc. Sci. Med..

[4] Panagioti M, Geraghty K, Johnson J, Zhou A, Panagopoulou E, Chew-Graham C, Peters D, Hodkinson A, Riley R, Esmail A (2018). Association between physician burnout and patient safety, professionalism, and patient satisfaction: A systematic review and meta-analysis. JAMA Intern. Med..

[5] McCrae RR, Costa PT, Hřebíčkovǎ M, Ostendorf F, Angleitner A, Avia MD, Sanz J, Sánchez-Bernardos ML, Kusdil ME, Woodfield R, Saunders PR, Smith PB (2000). Nature over nurture: Temperament, personality, and life span development. J. Pers. Soc. Psychol..

[6] Digman J (1990). Personality structure: Emergence of the five-factor model. Annu. Rev. Psychol..

[7] Bibbey A, Carroll D, Roseboom TJ, Phillips AC, de Rooij SR (2013). Personality and physiological reactions to acute psychological stress. Int. J. Psychophysiol..

[8] Ferguson E (2008). Health anxiety moderates the daytime cortisol slope. J. Psychosom. Res..

[9] Jonassaint CR, Why YP, Bishop GD, Tong EM, Diong SM, Enkelmann HC, Khader M, Ang J (2009). The effects of Neuroticism and Extraversion on cardiovascular reactivity during a mental and an emotional stress task. Int. J. Psychophysiol..

[10] Kirschbaum C, Prussner JC, Stone AA, Federenko I, Gaab J, Lintz D, Schommer N, Hellhammer DH (1995). Persistent high cortisol responses to repeated psychological stress in a subpopulation of healthy men. Psychosom. Med..

[11] Ó Súilleabháin PS, Howard S, Hughes BM (2018). Openness to experience and adapting to change: Cardiovascular stress habituation to change in acute stress exposure. Psychophysiology.

[12] Schneider TR (2004). The role of neuroticism on psychological and physiological stress responses. J. Exp. Soc. Psychol..

[13] Soliemanifar O, Soleymanifar A, Afrisham R (2018). Relationship between personality and biological reactivity to stress: A review. Psychiatry Investig..

[14] Williams PG, Rau HK, Cribbet MR, Gunn HE (2009). Openness to Experience and stress regulation. J. Res. Pers..

[15] Xin Y, Wu J, Yao Z, Guan Q, Aleman A, Luo Y (2017). The relationship between personality and the response to acute psychological stress. Sci. Rep..

[16] Everaerd D, Klumpers F, Wingen VG, Tendolkar I, Ferandez G (2015). Association between neuroticism and amysdala responsivitu emrges under stressful conditions. NeuroImage.

[17] Dias R, Neto A (2016). Stress levels during emergency care: A comparison between reality and simulated scenarios. J. Crit. Care.

[18] Krage R (2014). Does individual experience affect performance during cardiopulmanory resuscitation with additional external distractors?. Anaesthesia.

[19] Krage R (2017). Relationship between non-technical skills and technical performance during cardiopulmonary resuscitation: Does stress have an influence?. Emerg. Med. J..

[20] Schlatter S (2022). Effects of relaxing breathing paired with cardiac biofeedback on performance and relaxation during critical simulated situations: A prospective randomized controlled trial. BMC Med. Educ..

[21] Sigwalt F (2020). Stress management training improves overall performance during critical simulated situations: A prospective randomized controlled trial. Anesthesiology.

[22] Lilot M (2018). Relaxation before debriefing during high-fidelity simulation improves memory retention of residents at three months: A prospective randomized controlled study. Anesthesiology.

[23] Sanfilippo F, Noto A, Foresta G, Santonocito C, Palumbo GJ, Arcadipane A, Maybauer DM, Maybauer MO (2017). Incidence and factors associated with burnout in anesthesiology: A systematic review. Biomed. Res. Int..

[24] Schlatter S, Guillot A, Schmidt L, Mura M, Trama R, Di Rienzo F, Lilot M, Debarnot U (2021). Combining proactive transcranial stimulation and cardiac biofeedback to substantially manage harmful stress effects. Brain Stimul..

[25] Schlatter S, Schmidt L, Lilot M, Guillot A, Debarnot U (2021). Implementing biofeedback as a proactive coping strategy: Psychological and physiological effects on anticipatory stress. Behav. Res. Ther..

[26] De Read R, Hooley J (2016). The role of expectancy and proactive control in stress regulation: A neurocognitive framework for regulation expectation. Clin. Psychol. Rev..

[27] Juster R, Perna A, Marin M, Sindi S, Lupien S (2012). Timing is everything: Anticipatory stress dynamics among cortisol and blood pressure reactivity and recovery in healthy adults. Stress.

[28] Pulopulos MM, DeWitte S, Vanderhasselt MA, DeRaedt R, Schiettecatte J, Anckaert E, Salvador A, Baeken C (2019). The influence of personality on the effect of iTBS after being stressed on cortisol secretion. PLoS ONE.

[29] Brunoni A (2013). Enhancement of affective processing induced by bifrontal transcranial direct current stimulation in patients with major depression. Neuromodulation.

[30] Peciuliene I, Perminas A, Gustainiene L, Jarasiunaite G (2015). Effectiveness of progressive muscle relaxation and biofeedback relaxation in lowering physiological arousal among students with regard to personality features. Procedia. Soc. Behav. Sci..

[31] DeWitte NAJ, Buyck I, Van Daele T (2019). Combining biofeedback with stress management interventions: A systematic review of physiological and psychological effects. Appl. Psychophysiol. Biofeedback.

[32] Goessl VC, Curtiss JE, Hofmann SG (2017). The effect of heart rate variability biofeedback training on stress and anxiety: A meta-analysis. Psychol. Med..

[33] Subhani AR, Kamel N, Saad MNM, Nandagopal N, Kang K, Malik AS (2018). Mitigation of stress: New treatment alternatives. Cogn. Neurodyn..

[34] VanDiest I, Verstappen K, Aubert AE, Widjaja D, Vansteenwegen D, Vlemincx E (2014). Inhalation/exhalation ratio modulates the effect of slow breathing on heart rate variability and relaxation. Appl. Psychophysiol. Biofeedback.

[35] Lazarus RS, Folkman S (1984). Stress, Appraisal, and Coping.

[36] Nyklíček I, Irrmischer M (2017). For whom does mindfulness-based stress reduction work? Moderating effects of personality. Mindfulness.

[37] Pardine P, Napoli A (1977). Personality correlates of successful biofeedback training. Percept. Motor Skills.

[38] Plaisant O, Courtois R, Réveillère C, Mendelsohn GA, John OP (2010). Validation par analyse factorielle du Big Five Inventory français (BFI-Fr). Analyse convergente avec le NEO-PI-R. Ann. Medico-Psychol..

[39] Gauthier J, Bouchard S (1993). Adaptation Canadienne-Française de la forme révisée du State-Trait Anxiety Inventory de Spielberger. Can. J. Behav. Sci..

[40] Spielberger C, Gorsuch R, Lushene P, Vagg P, Jacobs A (1983). Manual for the State Trait Anxiety Inventory (Form Y).

[41] Childre D, Martin H (1999). The HeartMath Solution.

[42] McCraty R, Atkinson M, Tomasino D, Bradley RT (2009). The coherent heart. Integral Rev..

[43] McCraty R, Zayas MA (2014). Cardiac coherence, self-regulation, autonomic stability and psychosocial well-being. Front. Psychol..

[44] Shaffer F, McCraty R, Zerr CL (2014). A healthy heart is not a metronome: An integrative review of the heart’s anatomy and heart rate variability. Front. Psychol..

[45] John O, Donahue E, Kentle R (1991). The Big Five Inventory-Versions 4a and 54.

[46] John O, Srivastava S (1999). The Big Five trait taxonomy: History, measurement, and theorical perspectives. Handbook of Personality: Theory and Research.

[47] Van Der Wal RAB, Bucx MJL, Hendriks JCM, Scheffer GJ, Prins JB (2016). Psychological distress, burnout and personality traits in Dutch anaesthesiologists. Eur. J. Anaesthesiol..

[48] Baumstarck K (2017). Assessment of coping: A new french four-factor structure of the brief COPE inventory. Health Qual. Life Outcomes.

[49] Mirnics Z, Heincz O, Bagdy G, Surányi Z, Gonda X, Benko A, Molnar E, Jakšić N, Lazary J, Juhasz G (2013). The relationship between the big five personality dimensions and acute psychopathology: Mediating and moderating effects of coping strategies. Psychiatr. Danub..

[50] Shewchuk RM, Elliott TR, Macnair-Semands RR, Harkins S (1999). Trait influences on stress appraisal and coping: An evaluation of alternative frameworks. J. Appl. Soc. Psychol..

[51] Watson D, Hubbard B (1996). Adaptational style and dispositional structure: Coping in the context of the five-factor model. J. Pers..

[52] Penley J, Tomaka J (2002). Associations among the Big Five, emotional responses, and coping with acute stress. Personality Individ. Differ..

[53] McCrae RR, John OP (1992). An introduction to the Five-Factor Model and Its Applications. J. Pers..

[54] Peña-Gómez C, Vidal-Piñeiro D, Clemente IC, Pascual-Leone Á, Bartrés-Faz D (2011). Down-regulation of negative emotional processing by transcranial direct current stimulation: Effects of personality characteristics. PLoS ONE.

[55] Van Der Wal RAB, Bucx MJL, Hendriks JCM, Scheffer GJ, Prins JB (2016). Work stress and satisfaction in relation to personality profiles in a sample of Dutch anaesthesiologists: A questionnaire survey. Eur. J. Anaesthesiol..

[56] Van Der Wal RAB, Wallage J, Bucx MJL (2018). Occupational stress, burnout and personality in anesthesiologists. Curr. Opin. Anaesthesiol..

