# Supplementary material for: Personality traits affect anticipatory stress vulnerability and coping effectiveness in occupational critical care situations
Source: Sci Rep. 2022 Dec 5;12:20965. doi: 10.1038/s41598-022-24905-z (PMC9722917; doi:10.1038/s41598-022-24905-z)
Supplement: Supplementary file 1 — Supplementary Information. [file 41598_2022_24905_MOESM1_ESM.docx]

**Supplementary material**

**Annexe 1: Scenarios**

| Scenario | | Topic | Summary of the briefing |
| --- | --- | --- | --- |
| Tamponade | | Intra hospital cardiac arrest caused by hemopericardium leading to tamponade and sudden cardiac arrest | You are called to see a man with acute dyspnea and hypotension occurring few days after cardiac surgery |
| Neonatal cardiac arrest | | Desaturation and severe bradycardia just after childbirth | You are called for neonatal desaturation in the delivery room |
| Amniotic fluid embolism | | Amniotic fluid embolism leading to cardiac arrest of a pregnant woman just before non-urgent caesarean section | You are going to care for a pregnant woman for a non-urgent caesarean section |
| Pacemaker dysfunction | | Pacemaker and implantable cardioverter defibrillators dysfunction during emergent surgery (while using electrocautery) | You are supervising an emergent general anesthetic for a patient who has a pacemaker and implantable cardioverter defibrillators |
| Compressive pneumothorax | Intra hospital transport complicated by a compressive pneumothorax and cardiac arrest | | You have to manage a transport of a polytraumatised patient with a pneumothorax from the critical care unit to the scanner |
| Empty bottle of oxygen and split respirator tubing | Intra hospital transport complicated by a lack of oxygen due to an empty O2-bottle and a split respirator tubing | | You have to manage a transport of an intubated patient from the critical care unit to the scanner |
| Hemothorax | Intra hospital transport complicated by a hemorrhagic shock due to a hemothorax | | You have to manage a transport of an intubated patient with a drained hemothorax from the scanner to the critical care unit |
| Clogged endotracheal tube | Intra hospital transport complicated by an obstruction of the endotracheal tube | | You have to manage a transport of an intubated patient with a severe pneumonia from the critical care unit to the scanner |
| Anaphylactic shock | Anaphylactic shock during a general anaesthesia | | You are supervising a general anaesthesia of a young woman for an ectopic pregnancy with a hemoperitoneum |
| Cardiogenic shock | Fulminant myocarditis leading to a cardiogenic shock | | You are called to see a young man with chest pain and hemodynamic disorder in the emergency unit |
| Pulmonary embolism | Pulmonary embolism in a patient without anticoagulation after major surgery revelated by a cardiac arrest with a right ventricular failure | | You are called for an unconsciousness patient after the first lift in the critical care unit |
| Septic shock | Pyelonephritis leading to a septic shock | | You are called to see a young woman in the emergency room having low blood pressure despite vascular filling |
| Cannot intubate, cannot ventilate | Be familiar with the difficult tracheal intubation algorithm. Cannot intubate, cannot ventilate | | You are called to manage a young man in the emergency room with a laryngeal dyspnea |
| Fiberoptic endotracheal intubation | Be able to manage an awake fiberoptic endotracheal intubation | | You have to manage a general anesthesia in a patient with predictable difficult intubation |
| Emergency caesarean section | Be familiar with the difficult intubation algorithm. Cannot intubate, but can ventilate | | You are going to care for a pregnant woman for an urgent caesarean section |
| Laryngospasm | How to deal with a laryngospasm during a general anesthesia | | You have to manage an anesthesia in a young man for hardware removal |
| Atrial fibrillation | How to manage a bad tolerated atrial fibrillation in a septic patient with pneumoniae | | You are called in the emergency room to evaluate a patient with a pneumoniae |
| Local anesthetic intoxication | How to deal with a local anesthetic intoxication leading to a cardiac arrest | | You are called to survey a locoregional anesthesia for a tibial surgery |
| Medication error | How to deal with a medication error leading to a serious adverse event in the operating room | | You are called to survey a spinal anesthesia in the operating room for an inguinal hernia surgery |

**Annexe 2: Personality construction and comparison with a larger cohort of students**

Plaisant and collaborators (2010), asked more than 2400 students to answered the FR-Big5 questionnaire. The majority of their sample were medical students (60%), following by psychologist (25%) and sociology, history-geography, math, law students. The overall construction of personality is similar: in our study A > C > O > E > N and in the study of Plaisant A > C > O ≃ E > N. As the estimate difference between personality scores is less than 8% for each trait, we assume that our sample is similar to other French students’ sample. Finally, the repartition and scores of our sample is also comparable to other cultures (i.e., Americans and Spanish students).

|  | Our study  (n=147 medical students) | Plaisant et al. study (n=2499 students) | Estimate difference |
| --- | --- | --- | --- |
| Openness to experience | 3.32 ± 0.54 | 3.2 ± 0.6 | 0.12 (≃ 2 %) |
| Conscientiousness | 3.75 ± 0.58 | 3.4 ± 0.6 | 0.35 (≃ 7 %) |
| Extraversion | 3.08 ± 0.86 | 3.2 ± 0.7 | - 0.12 (≃ 2 %) |
| Agreeableness | 4.14 ± 0.53 | 3.8 ± 0.5 | 0.34 (≃ 7 %) |
| Neuroticism | 2.77 ± 0.78 | 2.9 ± 0.8 | - 0.13 (≃ 3 %) |
